# Innate immunity activation involved in unprotected porcine auto-transplant kidneys preserved by naked caspase-3 siRNA

**DOI:** 10.1186/1479-5876-11-210

**Published:** 2013-09-13

**Authors:** Cheng Yang, Long Li, Yinjia Xue, Zitong Zhao, Tian Zhao, Yichen Jia, Ruiming Rong, Ming Xu, Michael L Nicholson, Tongyu Zhu, Bin Yang

**Affiliations:** 1 Department of Urology, Zhongshan Hospital, Fudan University; Shanghai Key Laboratory of Organ Transplantation, 180 Fenglin Road, Shanghai 200032, PR China; 2 Transplant Group, Department of Infection, Immunity and Inflammation, University of Leicester; Leicester General Hospital, University Hospitals of Leicester, Gwendolen Road, Leicester LE5 4PW, UK; 3 Department of Nephrology, Affiliated Hospital of Nantong University, Nantong, PR China; 4 Department of Transfusion, Zhongshan Hospital, Fudan University, Shanghai, PR China; 5 Qingpu Branch Zhongshan Hospital, Fudan University, Shanghai, PR China

**Keywords:** Small interfering RNA, Innate immunity, Toll like receptor, PKR, Porcine kidney auto-transplantation, Ischemia reperfusion injury

## Abstract

**Background:**

The naked caspase-3 small interfering RNA (siRNA) infused into the renal artery during cold preservation was effective, but did not protect auto-transplant porcine kidneys with increased inflammation and apoptosis in our previous study. The mechanisms involved, in particular, whether siRNA or complementary systemic feedback eliciting innate immune responses are worthy to be further investigated.

**Methods:**

The protein and mRNA expression of innate immunity-related molecules were detected by western blotting and quantitative PCR in the tissues previously collected from 48 h auto-transplant kidneys. The donor kidneys were retrieved from mini pigs and cold preserved by University of Wisconsin solution with/without 0.3 mg caspase-3 siRNA for 24 h.

**Results:**

The protein level of Toll like receptor (TLR) 3, TLR7, and their main adapters, TRIF and MyD88, was up-regulated in the siRNA preserved auto-transplant kidneys. The mRNA level of NF-κB and c-Jun was increased, as well as pro-inflammatory cytokines, including IL-1β, IL-6, TNF-α and interferon (IFN)-α, β and γ. In addition, the non-TLR RNA sensor PKR protein, but not RIG1, was also increased in the siRNA preserved auto-transplant kidneys.

**Conclusions:**

The activation of innate immunity with amplified inflammatory responses in the caspase-3 siRNA preserved auto-transplant kidneys are associated with increased TLR3, TLR7 and PKR, which might be due to complementary systemic feedback, although persistent actions initiated by short-acting caspase-3 siRNA cannot be completely ruled out. These results provided valuable evidence to guide future siRNA design and pre-clinic studies.

## Background

Caspase-3 is a key enzyme that involves in the activation of inflammatory mediators and the execution of apoptosis. In our previous study, naked synthetic caspase-3 siRNA delivered into the isolated porcine kidney and hemoperfusate during cold preservation improved ischemia reperfusion injury (IRI) in post-reperfused kidneys [1]. The naked siRNAs supposed to be more suitable for acute disease such as IRI in transplantation and did not typically activate immune responses [2,3] because of its rapid degradation. Moreover, the treatment of recipient could be avoided, if the local administration of siRNA during donor preservation is enough to protect the transplant kidney. Therefore, the naked caspase-3 siRNA was infused into the renal artery during cold preservation, in which the caspase-3 siRNA was effective in terms of reducing caspase-3 mRNA and protein, but did not protect the siRNA preserved auto-transplant kidneys after 48 h, even increased caspase-3 expression, inflammation, apoptosis and renal tissue damage [4]. However, the mechanisms involved are not clear and worthy to be further investigated.

The RNA-sensing pattern recognition receptors (PRRs) are the most important components in the innate immunity. The response of PRRs to siRNA is mediated by either Toll like receptors (TLR) or non-TLR [5]. The RNA-sensing TLR (TLR3 and TLR7, their main adapters TRIF and MyD88) related to c-Jun [6] are predominantly located inside the cells. Non-TLR-mediated responses are triggered by siRNA binding to proteins such as dsRNA-binding protein kinase (PKR) associated with caspase-3, retinoic acid inducible gene 1 (RIG1) and NF-κB within the cytoplasm. TLR or non-TLR PRRs activation induces excessive cytokine release and inflammation [6]. In addition, high-mobility group box 1 protein (HMGB1) regulates gene expression acting as a nuclear transcription factor after cytoplasmic-nuclear translocation [7], which also linked to caspase-3 activation, apoptosis and inflammation [8].

Hence, it has been hypothesized that the naked caspase-3 siRNA itself activated PRRs via the TLR-dependent or non-TLR-dependent pathway and / or subsequent systemic complementary responses stimulated innate immunity, which then exacerbated the injury in auto-transplant kidneys.

## Methods

### Caspase-3 siRNA

Three pairs of siRNA, targeting porcine caspase-3 mRNA (NCBI CoreNucleotide Accession No.AB029345), were designed (Life Technologies, Paisley, UK). The most effective sequences: 5′-GGGAGACCUUCACAAACUUtt-3′ and 5′-AAGUUUGUGAAGGUCUCCCtg-3′, were selected in LLC-PK1 cells [9]. The *in vivo* ready custom caspase-3 siRNA (Silencer®) with selected sequences and optimized dosage were then verified in our *ex vivo* porcine kidney preservation study [1].

### Animals

Under the regulation laid down by the Chinese animal welfare authority, male mini pigs weighing 25-30 kg were used. They were housed with air condition, straw saw dust beds, and free access to water and fed with wetted granulated full fodder. All animal experiments were performed with the approval from the Laboratory Animal Ethical Committee of Fudan University.

### Anesthetic protocol

The animals were premedicated with 0.5 mg/kg of diazepam and 5 mg/kg of ketamine hydrochloride intramuscularly, followed by general anesthesia using 1 mg/kg of propofol (Fresenius Kabi, Bad Homburg, Germany) intravenously (i.v.), and maintained with a mix solution of 0.25 mg/kg/h of diazepam, 2.5 mg/kg of ketamine hydrochloride, and 0.0125 mL/kg/h of compound detomidine hydrochloride or 0.5 mg/kg/h of propofol i.v. in turn. The respiration was supported by a ventilator (Dräger, Lübeck, Germany) through an inserted trachea cannula. Five hundred milliliters of 5% glucose and 0.9% sodium chloride and 500 mL of hydroxyethyl starch 130/0.4 and sodium chloride injection (Fresenius Kabi, Bad Homburg, Germany) were also administered i.v. In addition, 100 mL of 0.3 g of levofloxacin lactate and 2 million units of benzylpenicillin were given i.v. 30 min before surgery. The same anesthetic protocol was used for donor retrieving and transplantation.

### Donor kidney retrieving and preservation

The left kidney was mobilized and removed with minimal warm ischemia (about 1 min) after ligating the renal artery near the abdominal aorta, renal vein near the inferior vena cava, and ureter. The isolated kidney was flushed immediately with 200 mL of precooled Ringer solution with 1000 IU of heparin at 100 cm H_2_O hydrostatic pressure until the kidney became pale and then followed by 200 mL of the University of Wisconsin (UW; Bristol-Myers Squibb, New York, NY) solution. At last, the half of 40 mL of precooled UW solution with (the treatment group, n = 5) or without (the negative control, n = 5) 0.3 mg of siRNA was infused into the renal artery to push out the remaining UW solution; the renal vein was then clamped and another 20 mL of the solution was infused, and the renal artery was finally clamped. The kidney was preserved on ice for 24 h.

### Right kidney nephrectomy and auto-transplantation

Next day, the right kidney was resected after ligating the right renal artery and vein, as well as the ureter, close to the renal hilum. The left kidney was orthotopically auto-transplanted into the right for 48 h. In addition, a double lumen cuffed silicone vascular access catheter (Arrow International, Reading, PA) was placed in the left internal jugular vein. The lumens of the central line were fixed behind the ear and blocked with heparin.

At 48 h post-transplant, the animal was anaesthetized, the blood sample was taken and sacrificed after harvested the graft. The part of renal tissues was fixed with 10% buffered formalin for histological examination and the others were snapping frozen for molecular biological analyses.

### Protein expression assay by western blotting

Twenty μg protein from kidney homogenate were separated on 15% (wt/vol) poly acrylamide denaturing gels and electro-blotted onto Hybond-C membranes. These membranes were blocked with 5% (wt/vol) milk, separately probed with anti-TLR3 (Cell Signaling Technology, Boston, USA), anti-TLR7 (LifeSpan BioSciences, Seattle, USA), anti-TRIF, anti-MyD88, anti-HMGB1, anti-RIG1, anti-PKR antibodies (all from Cell Signaling Technology, at 1:1,1000 dilution). For the loading control, the same membranes were probed with anti-β-actin antibody (1:10,000 dilution, Abcam, Cambridge, UK), then incubated with peroxidase-conjugated secondary antibodies (1:10,000 dilution, Jackson ImmunoResearch, West Grove, USA) at room temperature for 1 h. Immunoreactive bands were visualized using ECL substrate (Thermo Fisher Scientific, Rockford, USA) and a Bio-Image Analysis System (Cell Biosciences, Inc., Santa Clara, USA). The semi-quantitative analysis results were expressed as optical volume density (OD × mm^2^) and normalized by β-actin for loading (AlphaView Software 3.3, Cell Biosciences, Inc.).

### Inflammatory cytokines and transcription factors mRNA

Total RNA was extracted from renal tissues with Trizol reagent (Invitrogen, Carlsbad, USA). 1 μg of total RNA was reverse transcribed into cDNA using a RevertAid™ First Strand cDNA Synthesis Kit (Fermentas, Glen Burnie, USA). Real-time quantitative PCR (qPCR) was performed using the SYBR *Premix Ex Taq* Kit (Takara Bio Inc., Otsu, Japan) in a MasterCycler RealPlex4 system (Eppendorf, Hamburg, Germany). After a hot start (30 seconds at 95°C), amplification was performed for 45 cycles (5 seconds at 95°C, 30 seconds at 55°C, 60 seconds at 72°C). The primers were listed in Table 1. The expression of mRNA normalized with β-actin were calculated against relative non-IR injured kidneys (randomly selected 6 out of 12 post-nephrectomy kidneys) using a 2^−ΔΔ Ct^ method.

**Table 1:** The sequences of the primers

**Gene name**		**Primer sequence**
β-actin	Sense	CTCGGTCAGGATCTTCATGAGG
	Antisense	TTCTACAATGAGCTGCGTGTGG
IL-1β	Sense	TCATCGTGGCAGTGGAGAAGC
	Antisense	TCTGGGTATGGCTTTCCTTAG
IL-6	Sense	ATCTGGGTTCAATCAGGAGAC
	Antisense	CTAATCTGCACAGCCTCGAC
TNF-α	Sense	AACCCTCTGGCCCAAGGA
	Antisense	GGCGACGGGCTTATCTGA
IFN-α	Sense	TGGTGCATGAGATGCTCCA
	Antisense	GCCGAGCCCTCTGTGCT
IFN-β	Sense	AGTGCATCCTCCAAATCGCT
	Antisense	GCTCATGGAAAGAGCTGTGGT
IFN-γ	Sense	CATGAACACCATCAAGGAACAAAT
	Antisense	TTTGAATCAGGTTTTTGAAAGCC
NF-κB	Sense	CCCATGTAGACAGCACCACCTATGAT
	Antisense	ACAGAGGCTCAAAGTTCTCCACCA
c-Jun	Sense	TATGAGGAACCGCATCGCTG
	Antisense	TAGCATGAGTTGGCACCCACTG

### Statistical analysis

Results are expressed as mean ± standard error of the mean (SEM). Normality tests were carried out and statistical analysis of the data was performed with the two-tailed independent t-test between two groups. The correlations between parameters were determined by linear correlation and multiple regression analyses using SPSS 18.0 software (SPSS Inc, Armonk, NY, USA). P < 0.05 was considered as statistically significant.

## Results

In order to show a whole profile of this study, the results from our previous experiments using the same model were summarized as the following: the expression of caspase-3 mRNA precursor and active subunit (measured by qPCR and western blotting) was decreased in the post-CS kidneys preserved by caspase-3 siRNA, but the auto-transplanted kidneys were not protected, in which increased caspase-3 mRNA and its active subunit, inflammation [myeloperoxidase (MPO)+ cell staining], apoptosis [*in situ* end labeling (ISEL) fragmented DNAs] and tubulointerstitial damage (TID, assessed by hematoxylin and eosin staining) were demonstrated, even though the renal function (serum creatinine) was not significantly changed (Table 2) [4].

**Table 2:** Renal function, histopathology and molecular biology parameters in the kidney

**Detected parameters**	**PostN**	**PostCS**	**PostCS+siRNA**	P value*	**PostTx**	**PostTx+siRNA**	P value*
Caspase-3 mRNA	Not detected	2.22 ± 0.64	0.41 ± 0.12	0.017	3.77 ± 1.08	12.16 ± 3.70	0.042
32 kD caspase-3	0.50 ± 0.08	2.33 ± 0.28	1.11 ± 0.14	0.005	0.94 ± 0.08	0.27 ± 0.04	0.002
17 kD caspase-3	0.09 ± 0.01	Non-detectable	Non-detectable		0.07 ± 0.02	0.20 ± 0.06	0.026
SCr (μmol/L)	37.5 ± 8.1	76.83 ± 9.49	80.67 ± 7.10	0.753	506.4 ± 122.2	659.2 ± 49.1	0.273
BUN (mmol/L)	2.8 ± 0.4	8.05 ± 1.20	8.32 ± 0.95	0.865	35.9 ± 7.8	51.2 ± 6.5	0.164
Score of TID/200× field	Not detected	9.71 ± 0.56	7.93 ± 0.84	0.093	9.94 ± 0.53	11.90 ± 0.21	0.011
Active caspase-3+ cells/400× field	0.01 ± 0.00	1.05 ± 0.15	0.32 ± 0.10	0.004	2.96 ± 0.66	6.83 ± 1.26	0.017
ISEL+ cells /400× field	0.02 ± 0.01	0.30 ± 0.06	0.36 ± 0.19	0.734	1.39 ± 0.29	6.06 ± 1.37	0.004
MPO+ cells /400× field	0.77 ± 0.34	0.06 ± 0.04	0.08 ± 0.05	0.921	15.92 ± 3.84	52.33 ± 13.31	0.028

*PostN*, post-nephretomy; *PostCS*, post cold storage; *PostTx*, post-transplant; *SCr*, serum creatinine; *BUN*, blood urine nitrogen; *TID*, tubulointerstitial damage; *ISEL*, *in situ* end-labeling; *MPO*, myeloperoxidase. Data were presented as Mean ± SEM. *: The difference calculated between the non-preserved and siRNA-preserved kidney. The caspase-3 mRNA in the non-transplanted (PostN) control kidney was defined as baseline control using a 2^−ΔΔ Ct^ method.

### TLR3 and TLR7 protein expression

**Figure 1:**
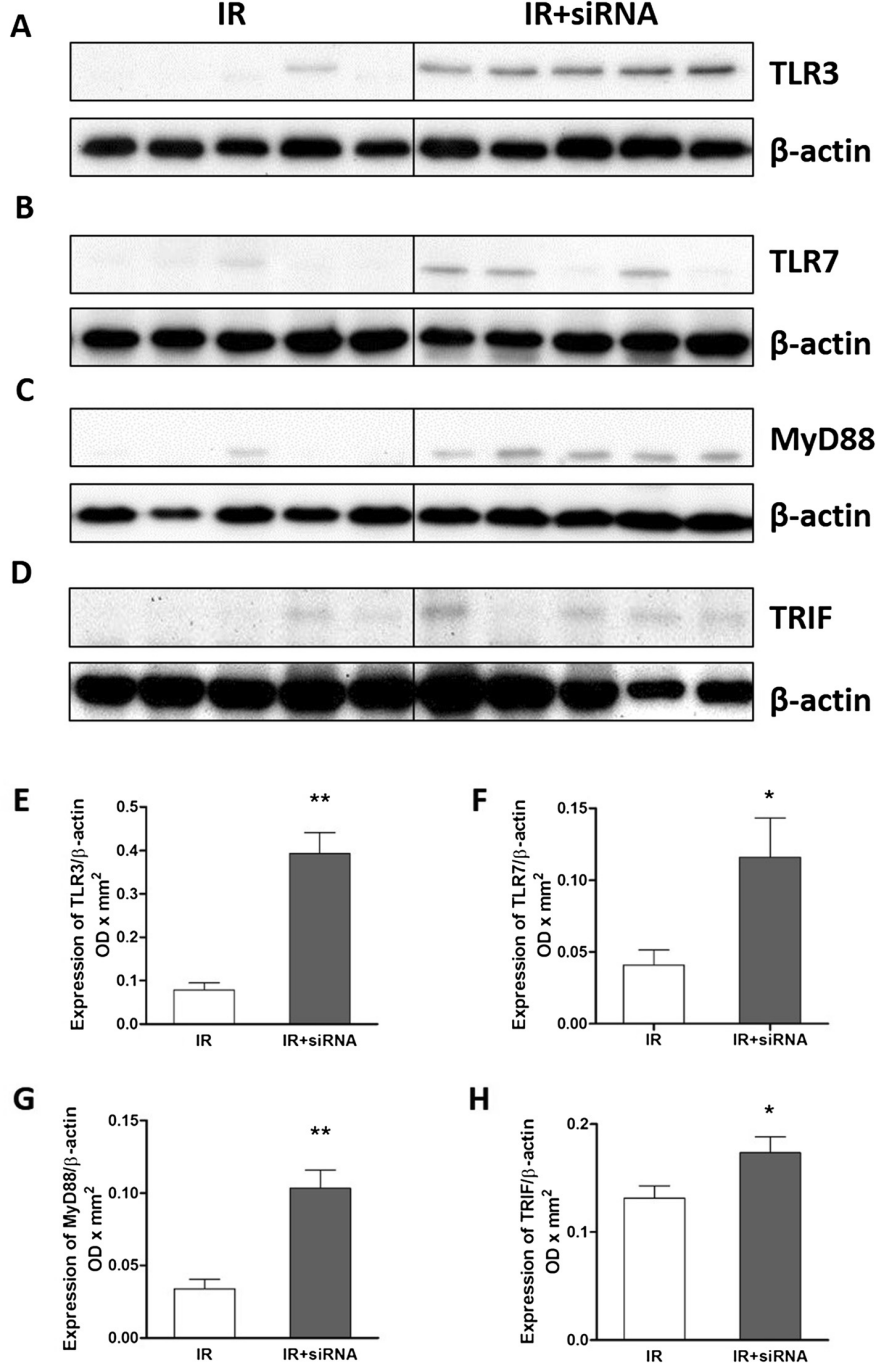
**TLR3, TLR7, MyD88 and TRIF protein in post-transplant kidneys.** The protein expression of TLR3, TLR7, MyD88 and TRIF detected by western blotting **(A**-**D)** was significantly increased in the caspase-3 siRNA preserved post-transplant kidneys **(E**-**H)**. The protein data are expressed as corrected volume density against the loading control of 42 kD β-actin. Mean ± SEM, n = 5. *: P < 0.05; **: P < 0.01.

To determine whether the TLR-dependent innate immunity was activated, the expression of TLR3, TLR7, MyD88 and TRIF protein in the post-transplant kidneys was detected by western blotting (Figure 1A-D). Semi-quantitative analysis revealed that TLR3, TLR7, MyD88 and TRIF were significantly increased in the siRNA preserved auto-transplant kidneys (0.39 ± 0.05 vs. 0.08 ± 0.02, 0.11 ± 0.04 vs. 0.05 ± 0.01, 0.10 ± 0.01 vs. 0.03 ± 0.01, 0.17 ± 0.02 vs. 0.12 ± 0.01, respectively, Figure 1E-H).

### PKR and RIG1 protein expression

**Figure 2:**
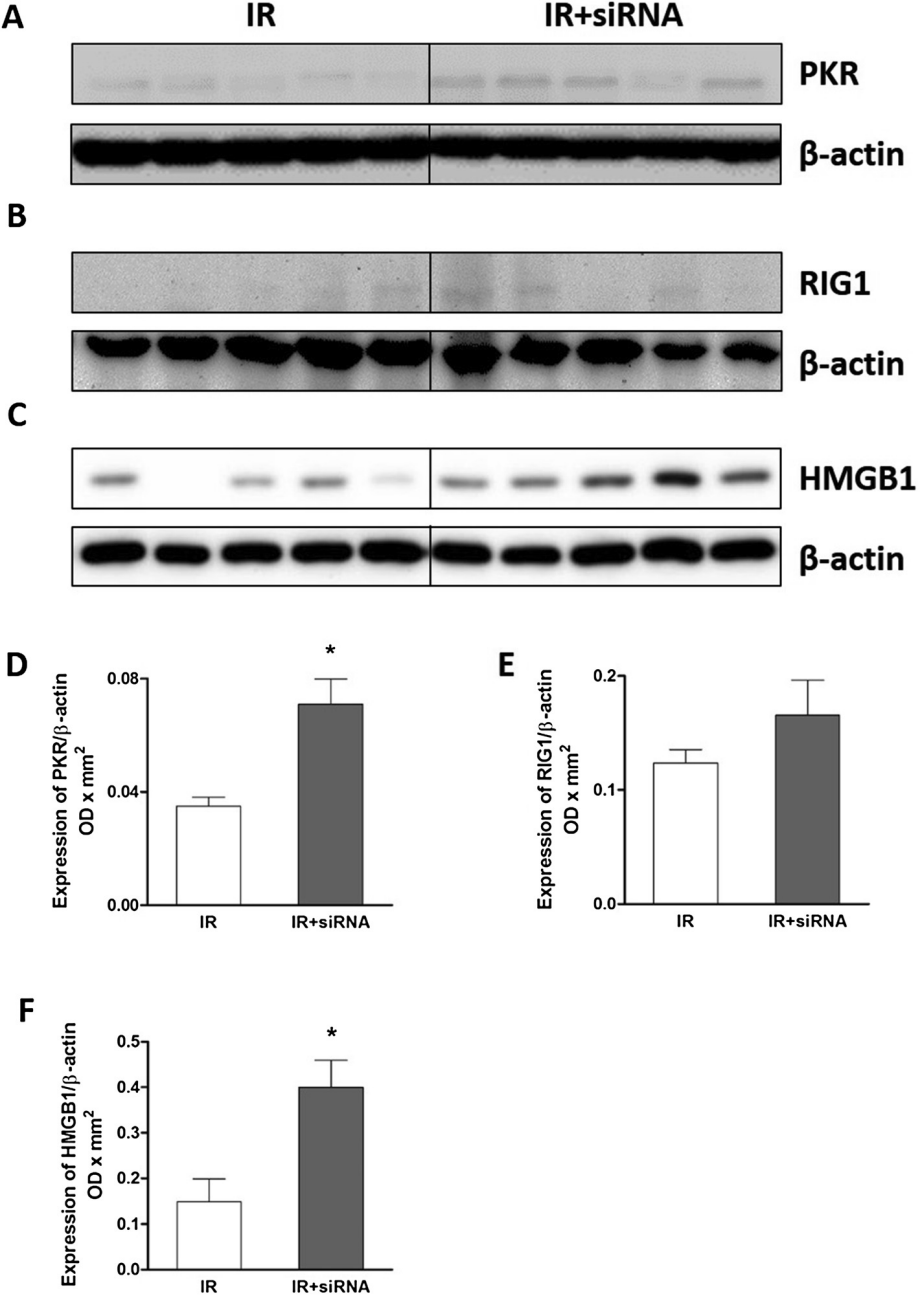
**PKR, RIG1 and HMGB1 protein in post-transplant kidneys.** The protein expression of PKR, RIG1 and HMGB1 detected by western blotting **(A**-**C)** was significantly increased in the caspase-3 siRNA preserved post-transplant kidneys **(D** and **F)**. There was no significant difference in RIG1 between the two groups **(E)**. The data are expressed as corrected volume density against the loading control of 42 kD β-actin. Mean ± SEM, n = 5. *: P < 0.05; **: P < 0.01.

To confirm whether the non-TLR-dependent innate immune response was also activated, the expression of RIG1 and PKR protein in the post-transplant kidneys was detected by western blotting as well (Figure 2A-B). In the siRNA preserved auto-transplant kidneys, the level of PKR was significantly increased (0.07 ± 0.01 vs. 0.03 ± 0.01, Figure 2D), while RIG1 protein was also up-regulated, but not reached significant differences between groups (Figure 2E).

### HMGB1 protein expression

The expression of HMGB1 protein, an important mediator of inflammation, was also evaluated by western blotting (Figure 2C). Semi-quantitative analysis revealed that HMGB1 was significantly increased in the siRNA preserved auto-transplant kidneys (0.40 ± 0.06 vs. 0.15 ± 0.05, Figure 2F).

### The mRNA level of pro-inflammatory cytokines and transcription factors

**Figure 3:**
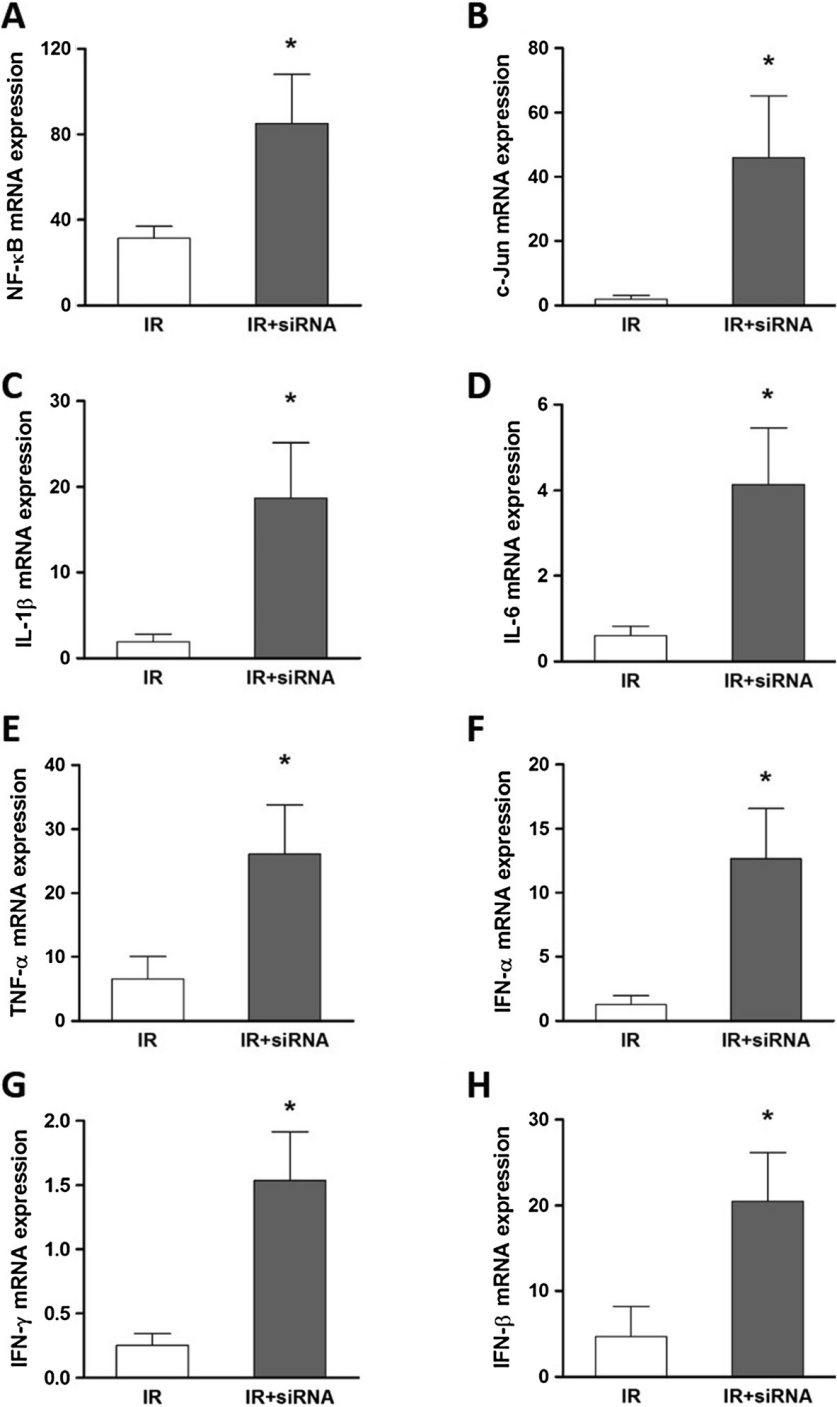
**The mRNA expression of inflammatory mediators in post-transplant kidneys.** The expression of IL-1β and IL-6, TNF-α, interferon (IFN)-α, IFN-β, IFN-γ, NF-κB and c-Jun detected by real-time qPCR was significantly increased in the caspase-3 siRNA preserved post-transplant kidneys **(A**-**H)**. The data are expressed as 2^−ΔΔ Ct^ normalized with β-actin relative to the normal kidneys of each group. Mean ± SEM, n = 5. *: P < 0.05; **: P < 0.01.

To check the downstream signaling events followed by the activation of TLR3, TLR7 and PKR, the mRNA expression of transcription factors, NF-κB and c-Jun, and pro-inflammatory cytokines, IL-1β, IL-6, TNF-α, IFN-α, IFN-β and IFN-γ was detected by real-time qPCR. Semi-quantitative analysis revealed NF-κB, c-Jun, IL-1β, IL-6, TNF-α, IFN-α, IFN-β and IFN-γ mRNA were all significantly increased by the preservation with caspase-3 siRNA (85.26 ±22.86 vs. 31.38 ± 5.56, 46.03 ± 19.17 vs. 1.98 ± 1.14, 18.68 ± 6.45 vs. 1.94 ± 0.90, 4.14 ± 1.31 vs. 0.60 ± 0.23, 26.13 ± 7.69 vs. 6.62 ± 3.47, 12.66 ± 3.94 vs. 1.27 ± 0.17, 20.49 ± 5.69 vs. 4.72 ± 3.46, 1.54 ± 0.38 vs. 0.25 ± 0.09, respectively, Figure 3A-H).

### Correlation between inflammation, apoptosis, HMGB1, TLR3, TLR7, PKR, renal function and structure

**Figure 4:**
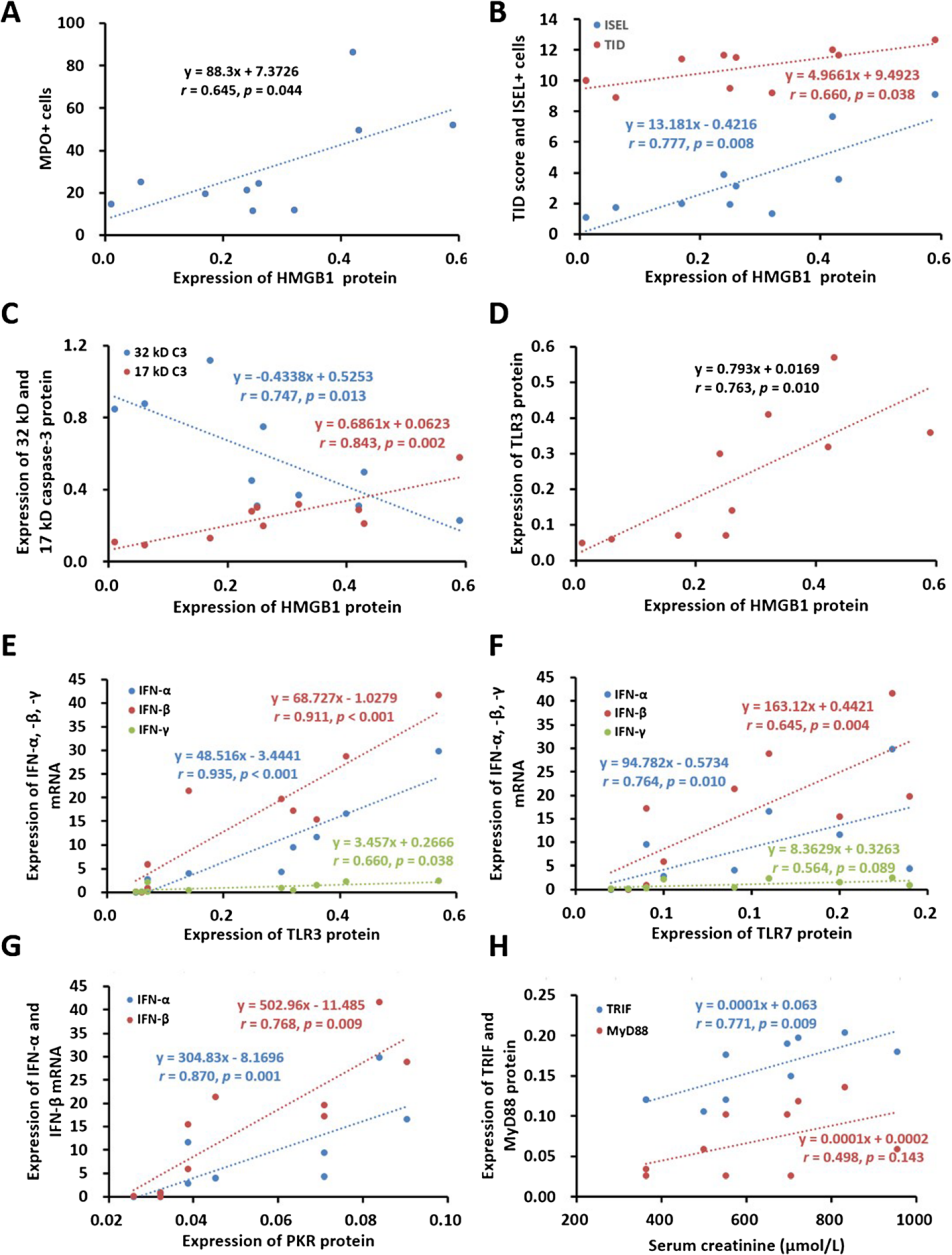
**Correlations between HMGB1, TLR3, TLR7, TRIF, PKR, caspase-3, inflammation, apoptosis, renal function and structure.** The positive correlations were revealed between HMGB1 protein and MPO+ cells **(A)**, TID score, ISEL+ cells **(B)**, 17 kD caspase-3 **(C)** and TLR3 **(D)**. The negative correlation was revealed between HMGB1 and 32 kD caspase-3 **(C)**. TLR3 **(E)**, TLR7 **(F)** and PKR **(G)** were also correlated with IFN-α, IFN-β and IFN-γ respectively. In addition, there were positive correlations between serum creatinine and TRIF or MyD88 **(H)**.

**Figure 5:**
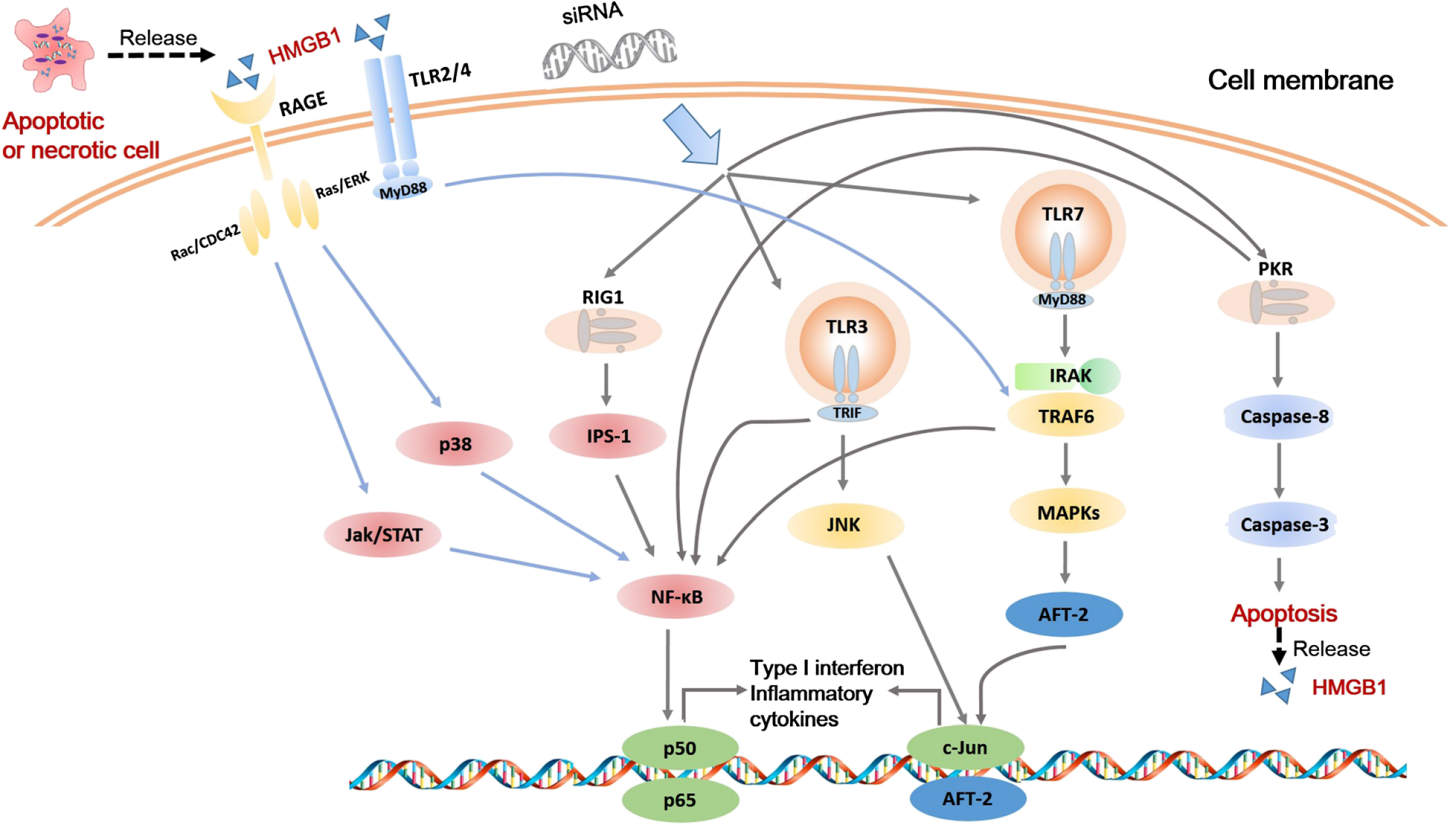
**A schematic illustration of the correlation in mechanisms among detected parameters.** Immune recognition of siRNA is via TLR pathway that mainly includes TLR3 (adaptor TRIF) and TLR7 (adaptor MyD88), as well as non-TLR pathway that involves PKR activation. NF-κB and c-Jun are located in convergent points of different signaling pathways and mediate interferons and inflammatory cytokines production. HMGB1 is released by apoptotic or necrotic cells, recognized by TLR2/4 or RAGE and associated with inflammatory responses.

In order to evaluate the relationship among inflammation, apoptosis and innate immune responses, the correlations between these parameters were analyzed. There were positive correlations between HMGB1 protein and MPO+, TID, ISEL+ cells (Figure 4A-B), 17 kD caspase-3 protein, as well as TLR3 protein (Figure 4C-D), but a negative correlation between HMGB1 protein and 32 kD caspase-3 protein was observed (Figure 4C). In addition, TLR3 or TLR7 protein was closely correlated with IFN-α, IFN- β or IFN-γ mRNA (Figure 4E), while PKR protein was only correlated with IFN-α and IFN- β mRNA (Figure 4F-G). There were also positive correlations between serum creatinine and TRIF or MyD88 protein (Figure 4H). A schematic illustration showed the correlation among detected parameters (Figure 5).

## Discussion

Our serial *in vitro*, *ex vivo* and *in vivo* studies used naked siRNA to minimize off-target effects, which were often induced by siRNA vehicles. The recognition of the naked caspase-3 siRNA duplexes as a foreign entity by the innate immune system acts as a trigger for the rapid release of inflammatory mediators as part of the natural defense of hosts against pathogens. The naked siRNA, however, has very short half-life in serum. In the caspase-3 siRNA preserved 48-h auto-transplanted kidneys, the expression of TLR3 and TLR7, their adaptors MyD88 and TRIF, as well as TLR-independent PKR, down-stream mediators NF-κB and c-Jun, IFN-α, β and γ, cytokines TNF-α, IL-1β and IL-6 were all up-regulated. These changes might be more likely ascribed to systemic complementary responses, although the persistent action initiated by caspase-3 siRNA cannot be completely ruled out.

Due to effectiveness of caspase-3 siRNA in kidney preservation, but easy degenerating property of naked siRNA, more caspase-3 proteins were synthesized and cleaved into active subunits in the 48-h auto-transplanted kidneys. In addition, the cellular stress upon reperfusion without systemic supplement of caspase-3 siRNA also could contribute to the enhanced caspase-3 expression. Although all possible aspects involved in unprotected auto-transplant kidney were well discussed in our previous publication [4], the activation of innate immunity and its possible pathways, as well as amplified inflammatory reactions, were further demonstrated in this study. These orchestrated cascade changes might be mainly initiated by the systemic feedback due to the low level of caspase-3 mRNA and protein in the caspase-3 siRNA preserved kidneys. The end results were not only more severe inflammation mellitus, but also more apoptotic cell death and renal tissue damage.

It has been shown that, TLR3, apart from TLR2 and TLR4, induces cellular apoptosis via the PI3K-Akt signaling pathway [10]. Endosomal TLR3 signaling occurs through the TRIF adaptor leading to IFN regulatory factor 3 activation and IFN-β expression. The TLR3 signaling also activates down-stream mediators NF-κB and c-Jun, which cause the production of inflammatory cytokines. TLR3 in the surface of endothelial cells could be also activated by exogenous siRNA, at least 21 nt in length, *in vivo*, leading to the production of cytokines such as IFN-γ and IL-12 [11]. The up-regulated TLR3 and TRIF were observed in the caspase-3 siRNA persevered auto-transplant kidneys, which indicate the activation of TLR3 signaling. However, it was very difficult to trace the location of caspase-3 siRNA and prove whether porcine TLR3 was directly activated by the caspase-3 siRNA in this study. It also needs to be taken into account that there is a great homology between porcine and human, whereas human TLR3 is not activated by siRNA due to the structure difference in the activation point [12].

In addition, the production of IFN-α and inflammatory cytokines could be triggered by synthetic siRNAs and short hairpin RNAs dose-dependently via TLR7 in immune cells including plasmacytoid dendritic cells (DCs) and myeloid DCs [3,13,14]. TLR7 is constitutively expressed in these cells [15]. Interestingly, TLR7 preconditioning mediates neuroprotection against ischemic injury and the mechanism involved is unique in contrast to other TLR preconditioning ligands, which dependents on type I IFN receptor, but is independent of TNF [16]. Consistently, the increased TLR7 was demonstrated in the caspase-3 siRNA preserved auto-transplant kidneys in this study, accompanied by MyD88 activation, which led to the activation and nuclear translocation of NF-κB and c-Jun. As a result, the production of IFN-α, IFN-β and IFN-γ, as well as inflammatory cytokines TNF-α, IL-1β and IL-6, were remarkably increased and auto-transplant kidney structure was more severely damaged [6,17-20]. These cascade responses more liked were due to a systemic feedback post 48-h transplantation, rather than persistent actions initiated by caspase-3 siRNA in 24-h cold preservation.

HMGB1 secretion and inflammation promote each other [21,22]. Both HMGB1 and infiltrated myeloperoxidase (MPO)+ neutrophils were significantly higher in siRNA preserved auto-transplant kidneys [4], which indicate MPO+ cells might be recruited by highly expressed HMGB1 protein locally. Combined with our previous data [4], the positive correlations between HMGB1 protein and MPO+ cells, inflammatory mediators, apoptosis or innate immune activation were revealed, which indicated HMGB1 might promote inflammation and apoptosis. A recent study also revealed that fewer neutrophils infiltrated in the myocardium of *tlr4* (HMGB1 receptor) mutant mice was observed after myocardial IR, and *tlr4* deficiency markedly decreased IRI with inhibited HMGB1, TNF-α, and IL-8 [21]. Furthermore, HMGB1 promotes neutrophil extracellular trap formation and may contribute to the severity of neutrophil-associated inflammatory conditions [23].

Interestingly, the close correlations were observed, between HMGB1 and caspase-3 protein, positive with 17 kD active subunit, but negative with 32 kD precursor. As we explained in the previous study [4], the feedback regulation induced by short-term caspase-3 silence caused more caspase-3 precursor to be cleaved into active subunits. Hence, more cells in the siRNA preserved auto-transplant kidneys underwent to apoptosis followed by HMGB1 secretion. With the siRNA treatment, the increased HMGB1 recruited more neutrophils, and produced more inflammatory cytokines, which then amplified inflammation and aggravated tissue lesion. In addition, HMGB1 expression was strong correlated with TLR3 expression, not TLR7 or PKR. TRIF, an adapter protein in TLR3 signal transduction, not MyD88, also correlated positively with serum creatinine. Therefore, it suggests that TLR3 activation together with increased TRIF might take main responsibility for renal tissue damage.

Recently, certain modulations of siRNAs have been developed to maintain its efficacy, but reduce unpleasant side effects. A novel chemical modified 21-mer epithelial sodium channel siRNA with 85% knockdown efficiency showed no evidence for potential to stimulate TLR3, TLR7 or TLR8 [24]. In addition, 8-Alkoxyadenosine phosphoramidites was incorporated into the guide strand of caspase-2 siRNA at different positions, in which single modifications at positions 6 and 10 were effective in blocking siRNA binding to the PKR and might reduce sequence-independent side effects [25]. The bifunctional siRNAs with both gene silencing and innate immune activation properties, however, may represent a new potential strategy for treatment of virus infection. A chemically synthesized HBVx siRNA not only inhibited HBVx mRNA expression, but also increased expression of PKR leading to the higher production of type I IFN [26].

It has been also noted that using a multi-targeted siRNA cocktail is a better approach than simply increasing the dose of the best performed single siRNA. The combinational siRNA strategy applying lower concentration of each siRNA may reduce the off-target effects without sacrificing silencing potency [27]. Such strategies have been tested in IRI treatment. For instance, the blockade of caspase-3 and caspase-8 [28] or caspase-3 and complement 3 [29] simultaneously in renal IRI were beneficial. A solution with combined siRNAs targeting TNF-α, complement 3 and Fas inhibited heart graft injury with prolonged graft survival [30]. Therefore, knocking down elements involving in TLR pathways together with caspase-3 might be more efficient to ameliorate IRI in transplantation, which is worthy to be further investigated.

## Conclusion

In conclusion, the locally administrated naked caspase-3 siRNA in preservation might be degenerated after transplantation without systemic supplement, as the complementary increase in caspase-3 mRNA and protein was detected in the auto-transplanted kidneys. The activation of innate immunity via TLR3, TLR7 and PKR associated with amplified inflammatory responses, increased interferons and pro-inflammatory cytokines in the auto-transplant kidneys were more liked due to the systemic complementary responses, although persistent actions initiated by the siRNA cannot be completely ruled out. In further studies, the sequence design, the stability, the delivery route and time of siRNA should be taken into account more intentionally before siRNA therapy to be translated from bench to bed.

## Abbreviations

IRI: Ischemia-reperfusion injury; siRNA: Small interfering RNA; CS: Cold storage; TLR: Toll like receptor; IFN: Interferon; PRRs: Pattern recognition receptors; RIG1: Retinoic acid inducible gene 1; PKR: dsRNA-binding protein kinase; HMGB1: High-mobility group box 1; qPCR: Quantitative PCR; TID: Tubulointerstitial damage; MPO: Myeloperoxidase; ISEL: *In situ* end labelling.

## Competing interests

The authors declare that they have no competing interests.

## Authors’ contributions

CY: Participated in the writing of the paper, animal surgery, the performance of the research and data analysis. LL: Participated in animal surgery, the western blot and PCR experiments. YX: Participated in the performance of the research and data analysis. ZZ: participated in the performance of the research and data analysis. TZ: Participated in animal surgery and the performance of the research. YJ: Participated in research design, preparation and the performance of the research. RR: participated in the performance of the research. MX: participated in the performance of the research. MLN: Participated in research design and support. TZ: Participated in research design and support. BY: Participated in research design, the performance of the research, data analysis and the writing of the paper. All authors read and approved the final manuscript.
